# 

**Published:** 1880-11

**Authors:** 


### Books and Pamphlets Received.

### BOOKS.

Treatise on Therapeutics. By Trousseau & Pidoux. Translated by Dr. D. T. Lincoln. Vol. I. New York: Wm. Woods & Co. 1880.

The Art of Prolonging Life. By C. W. Hufeland.

What to Do First in Accidents or Poisoning. By C. W. Dulles, M.D.

Lessons in Laryngoscopy, and Rhinoscopy, and the Diagnosis and Treatment of Throat Diseases. By Prosser James, M.D.

A Treatise on the Practice of Medicine. By Roberts Bartholow, M.D. Pp. 853, cloth. New York: D. Appleton & Co.; 1880.

On the Bile, Jaundice and Bilious Diseases. By J. Wickham Legg.

A Treatise on the Diseases of the Eye. By J. Soelberg Wells, M.D. Philadelphia: A. C. Lea's Son & Co.; 188[illegible].

### PAMPHLETS.

Eighth Annual Report Michigan State Board of Health; 1880.

Twenty-third Annual Report Missouri Medical Association; 1880.

National Association for Protection of Insane, and Prevention of Insanity

Transactions Southern Illinois Medical Association for 1880; Vol. II.

Transactions California Medical Society for 1878–79–80.

Indications for Treatment in Fractures at Elbow. By L. S. Pilcher, M.D.

A Contribution to Pathological Histology of Acute Parotitis. By E. Croendt, M.D.

Transactions Kansas State Medical Society for 1880.

Rise of American Dermatology. By L. A. Duhring, M.D. New York: A. G. Sherwood & Co. 1880.

Contribution to Knowledge of Fracture of Rim of Acetabulum; twenty-seven cases. By N. Senn, M.D. 1880.

Practical Suggestions in Treatment of Diphtheria. By R. J. Nunn, M.D., Savannah, Georgia.

Presentation of the Funis, with notes of ten cases. By J. G. Meachem, Jr., M.D., of Racine, Wisconsin.

Hernia in Children; based on a study of 500 cases. By Edw. Swasey, M.D.

Transactions Georgia Medical Association for 1880.

---

THE DIABETOMETER. — A method for the accurate and easy estimation of the quantity of sugar contained in a given quantity of diabetic urine is very desirable. M. Yvon in the *Bulletin Générale de Thérapeutique*, for July 16, figures and describes an instrument which seems to fulfill all the indications, and if accurate, should prove a boon to the practical physician, and should certainly find a place in all large hospitals. It consists essentially of a glass tube, intended to hold the urine to be used, and which is supported horizontally on a stand. In the end of this nearest the source of light are placed, first, a reservoir for bicarbonate of potassium, then a polarizer. At the other end of the glass tube is a Nichols prism, and then a pair of lenses to focus its ray of light on the eye. The Nichols prism is made to rotate by means of a milled head, graduated so as to express the richness of sugar in grams to the liter of urine.—*Phila. Med. Times.*

CHARMS.—Stones were up to a recent period of civilization used as amulets, charms, tests and cures in both mental and bodily ailments. The blood-stone was used to stop blood running from wounds; the jacinth for driving away fever and dropsy; the moonstone was venerated from its supposed lunar attraction; coral kept off the evil eye; amber was the amulet against insanity, and also cured the ague. But the list is too long for our space. See Rymer, "Fœdera," Vol. I., page 139. —*Medical Times and Gazette.*

SOCIETY MEETINGS.

Chicago Medical Society—Mondays, Nov. 1 and 15.

West Chicago Medical Society—Mondays, Nov. 8 and 22.

Biological Society—Wednesday, Nov. 3.

CLINICS.

MONDAY.

Eye and Ear Infirmary—2 p. m., Ophthalmological, by Prof. Holmes; 3 p. m., Otological, by Prof. Jones.

Mercy Hospital—2 p. m., Surgical, by Prof. Andrews.

Rush Medical College—2 p. m., Dermatological and Venereal, by Prof. Hyde.

Woman's Medical College—2 p. m., Dermatological and Venereal, by Prof. Maynard; 3 p. m., Diseases of the Chest, Prof. Ingals.

TUESDAY.

Cook County Hospital—2 to 4 p. m., Medical and Surgical Clinics.

Mercy Hospital—2 p. m., Medical, by Prof. Quine.

WEDNESDAY.

Chicago Medical College—2 p. m., Eye and Ear, by Prof. Jones.

Rush Medical College—2 p. m., Medical, by Dr. Bridge; 3 p. m., Ophthalmological and Otological, by Prof. Holmes; 3:30 to 4:30 p. m., Diseases of the Chest, by Dr. E. Fletcher Ingals.

THURSDAY.

Chicago Medical College—2 p. m., Gynæcological, by Prof. Jenks.

Rush Medical College—2 p. m., Diseases of Children, by Dr. Knox; 3 p. m., Diseases of the Nervous System, by Prof. Lyman.

Eye and Ear Infirmary—2 p. m., Ophthalmological, by Dr. Hotz.

Woman's Medical College—3 p. m., Surgical, by Prof. Owens.

FRIDAY.

Cook County Hospital—2 to 4 p. m., Medical and Surgical Clinics.

Mercy Hospital—2 p. m., Medical, by Prof. Davis.

SATURDAY.

Rush Medical College—2 p. m., Surgical, by Prof. Gunn; 3 p. m., Orthopœdic, by Prof. Owens.

Chicago Medical College—2 p. m., Surgical, by Prof. Isham; 3 p. m., Neurological, by Prof. Jewell.

Woman's Medical College—11 a. m., Ophthalmological, by Prof. Montgomery; 2 p. m., Gynæcological, by Prof. Fitch.

Daily Clinics, from 2 to 4 p. m., at the Central Free Dispensary, and at the South Side Dispensary.

